# CDK9 Inhibitor Induces the Apoptosis of B-Cell Acute Lymphocytic Leukemia by Inhibiting c-Myc-Mediated Glycolytic Metabolism

**DOI:** 10.3389/fcell.2021.641271

**Published:** 2021-03-04

**Authors:** Wen-Li Huang, Tuersunayi Abudureheman, Jing Xia, Lei Chu, Hang Zhou, Wei-Wei Zheng, Neng Zhou, Rong-Yi Shi, Ming-Hao Li, Jian-Min Zhu, Kai Qing, Chao Ji, Kai-Wei Liang, Sa Guo, Gang Yin, Cai-Wen Duan

**Affiliations:** ^1^Department of Pathology, School of Basic Medical Science, Central South University, Changsha, China; ^2^Department of Pathology, The Affiliated Hospital of Youjiang Medical University for Nationalities, Baise, China; ^3^Key Laboratory of Pediatric Hematology and Oncology, Shanghai Children’s Medical Center, Ministry of Health, Pediatric Translational Medicine Institute, Shanghai Jiao Tong University School of Medicine, Shanghai, China; ^4^Department of Gynecology and Obstetrics, Shanghai Tongji Hospital, Tongji University School of Medicine, Shanghai, China; ^5^Shanghai Collaborative Innovation Center for Translational Medicine, Department of Pharmacology and Chemical Biology, Shanghai Jiao Tong University School of Medicine, Shanghai, China; ^6^State Key Laboratory of Medical Genomics, National Research Center for Translational Medicine at Shanghai, Shanghai Institute of Hematology, Ruijin Hospital Affiliated to Shanghai Jiao Tong University School of Medicine, Shanghai, China; ^7^Department of Pathophysiology, School of Basic Medical Sciences, Wuhan University, Wuhan, China

**Keywords:** CDK9 inhibitors, cell apoptosis, glycolysis, c-Myc, B-cell acute lymphocytic leukemia

## Abstract

B-cell acute lymphocytic leukemia (B-ALL), a common blood cancer in children, leads to high mortality. Cyclin-dependent kinase 9 inhibitor (CDK9i) effectively attenuates acute myeloid leukemia and chronic lymphoblastic leukemia by inducing apoptosis and inhibiting cell proliferation. However, the effect of CDK9i on B-ALL cells and the underlying mechanisms remain unclear. In this study, we showed that CDK9i induced the apoptosis of B-ALL cells *in vitro* by activating the apoptotic pathways. In addition, CDK9i restrained the glycolytic metabolism of B-ALL cells, and CDK9i-induced apoptosis was enhanced by co-treatment with glycolysis inhibitors. Furthermore, CDK9i restained the glycolysis of B-ALL cell lines by markedly downregulating the expression of glucose transporter type 1 (GLUT1) and the key rate-limiting enzymes of glycolysis, such as hexokinase 2 (HK2) and lactate dehydrogenase A (LDHA). Moreover, cell apoptosis was rescued in B-ALL cells with over-expressed c-Myc after treatment with CDK9i, which is involved in the enhancement of glycolytic metabolism. In summary, our findings suggest that CDK9 inhibitors induce the apoptosis of B-ALL cells by inhibiting c-Myc-mediated glycolytic metabolism, thus providing a new strategy for the treatment of B-ALL.

## Introduction

B-cell acute lymphoblastic leukemia (B-ALL) is one of the most frequently occurring malignancies in children, with a peak incidence between 1 and 4 years of age. Considering the improvements in multimodal chemotherapy regimens over the past few decades, the 5-year survival rate for pediatric B-ALL is now close to 90% (Malard and Mohty, 2020). However, a proportion of patients still shows no response to existing therapeutic drugs and suffered from the side-effects of long-term multi-drug treatment. In addition, existing therapeutic drugs cannot further improve the prognosis of refractory and relapsed B-ALL (Kuhlen et al., 2019). Therefore, new strategies for the treatment of B-ALL should be identified.

The inhibition of the cell cycle is one of the key mechanisms in the development of drugs for leukemia treatment (Ghelli Luserna di Rora et al., 2017). Therefore, chemotherapeutic drugs mainly interfere with DNA synthesis and inhibit cell cycle on leukemic cells. Cyclin-dependent kinases (CDKs) are one family of serine/threonine protein kinases and regulate the cell cycle division and gene transcription. CDKs can be divided into two categories according to their function, namely, CDKs that regulate cell cycle and CDKs that modulate gene transcription (Malumbres, 2014; Lemmens and Lindqvist, 2019). Cyclin-dependent protein 9 (CDK9) belongs to the CDK cyclin family, which includes in CDK4, CDK6, and CDK7. CDK9 modulates the transcription elongation and mRNA maturation of genes but does not regulate cell cycle of cells (Asghar et al., 2015). CDK9 phosphorylates Ser-2 and Ser-5 of the carboxyl terminal domain (CTD) of RNA polymerase II (RNA Pol II), which is involved in transcription elongation (Laitem et al., 2015; Gressel et al., 2017). CDK9 participates in the development and progression of many types of tumors by recruiting p-TEFb to the promoters of oncogenes in a BRD4-dependent manner (Franco et al., 2018). Therefore, CDK9 could serve as a potential therapeutic target in most of malignant tumors (Sonawane et al., 2016). CDK9 inhibitors have a significant inhibitory effect on acute myeloid leukemia (AML) and chronic lymphocytic leukemia (CLL) (Yin et al., 2014; Boffo et al., 2018). SNS-032, a CDK9 selective inhibitor, has entered clinical trials for the treatment of AML, CLL, and multiple myeloma (Tong et al., 2010; Walsby et al., 2011). AZD4573, another highly selective inhibitor of CDK9, has been validated in hematological malignancies (Cidado et al., 2020). However, the effect of CDK9 inhibitors on B-ALL cells and the underlying mechanism remain unknown.

Tumor cells favor anaerobic glycolysis as energy source even under sufficient oxygen condition, which is known as the Warburg effect. As the initial step in glucose metabolism, glycolysis consists of several reactions that are involved in several key rate-limiting enzymes, such as hexokinase (HK), phosphofructokinase (PFK), and pyruvate kinase (PK) (Counihan et al., 2018). CDK6 links the cell cycle and cell metabolism of tumors by phosphorylating two key enzymes, 6-phosphofructokinase (PFK1), and pyruvate kinase M2 (PKM2) and leads to the inhibition of glycolytic pathway and fuels the pentose phosphate (PPP) and serine pathways (Wang et al., 2017). CDK9 inhibition stops the gene transcription and results in the downregulated expression of a large proportion of genes, such as c-Myc and Mcl-1 (Boffo et al., 2018). The oncogene c-Myc controls many aspects of cell biological processes, such as cell growth, proliferation, differentiation, and apoptosis (Garcia-Gutierrez et al., 2019). As a metabolic sensor, c-Myc stimulates the glycolysis, mitochondrial biogenesis and glutamine metabolism by directly modulating the expression of metabolism-related genes in tumor cells (Stine et al., 2015; Dejure and Eilers, 2017). However, whether CDK9 inhibitors induce cell apoptosis in leukemia by suppressing c-Myc-mediated glycolysis is largely unknown.

The oncogene c-Myc encodes a transcription factor c-Myc, which links altered cellular metabolism to tumorigenesis. c-Myc regulates genes involved in the biogenesis of ribosomes and mitochondria, and regulation of glucose and glutamine metabolism.

In the study, we discovered that CDK9 inhibitors induced the apoptosis of B-ALL cells by restraining glycolysis, which was enhanced by co-treatment with glycolysis inhibitors *in vitro*. Moreover, cell apoptosis was reversed in B-ALL cells with overexpressed c-Myc after treatment with CDK9 inhibitors, which are involved in the enhancement of glycolytic metabolism. Therefore, these findings provide a potential treatment strategy for B-ALL in the clinic.

## Materials and Methods

### Clinical Samples

Bone marrow samples from patients with childhood B-ALL were collected in Shanghai Children’s Medical Center (SCMC). Sample usage and protocols were approved and supervised by the SCMC Ethics Committee. All the samples were analyzed in a blind manner and stored in SCMC. B-ALL cells were seeded at a density of 10^6^ cells/ml in STEMSPAN (Gibco) medium supplemented with 20 ng/ml recombinant human IL3 (rhIL3), 10 ng/ml rhIL7, 10 ng/ml rhIL6, 10 ng/ml rhIL2, 10 ng/ml rhIGF-1, 20 ng/ml rhFlt3L, and 10 ng/ml rhVcam1. B-ALL cells were treated with or without 1 μM of SNS-032 for 24 h, and the percentage of apoptosis was analyzed by flow cytometry.

### Culture of Cell Lines

Human B-ALL cell lines, SEM, RS4;11, NALM6, and REH, were purchased from the American Type Culture Collection (Manassas, VA, United States) and cultured in RPMI-1640 medium supplemented with 10% fetal bovine serum (FBS, Gibco) and 1% penicillin–streptomycin (Gibco). Cell lines were routinely detected by mycoplasma contamination test and were assessed using short tandem repeat (STR) DNA profiling.

### Drug Sensitivity Assay

A total number of 12,000 cells per well were seeded in a 96-well plate and then treated with different concentration of drugs (SNS-032 and AZD4573, obtained from Selleck, Houston, TX, United States) for 72 h. Cell viability was evaluated using CTG (Promega CellTiter-Glo^TM^ Luminescent Cell Viability Assay Kit) according to the manufacturer’s protocol. The absorbance optical density of 405 nm was recorded using a microplate reader (Synerge2; BioTek Instruments, Winooski, VT, United States), and the half maximal inhibitory concentration (IC50) was calculated using GraphPad Prism.

### Quantitative Reverse Transcription–Polymerase Chain Reaction (qRT-PCR)

Total RNA was isolated using the TRIzol reagent (Life technologies). qRT-PCR was performed according to the protocol of QIAGEN SYBR Green PCR. The primers are listed as follows: β-actin: forward (F): 5′-TGCCGACAGGATGCAGAAG-3′ and reverse (R): 5′-GCCGATCCACACGGAGTACT-3′; HK2: F: 5′-CTCTCTGCAACCAGTTCTCTG-3′ and R: 5′-CCAGGCAT TCGGCAATGTG-3′; LDHA: F: 5′-ATGGCAACTCTAAAGGA TCAGC-3′ and R: 5′-CCAACCCCAACAACTGTAATCT-3; BCL2: F: 5′-AAGATTGATGGGATCGTTGC-3′ and R: 5′-TGT GCTTTGCATTCTTGGAC-3′; GLUT1: F: 5′-CAGTTTGTGC TTTGCTGGCTACAACACTGGAGT-3′ and R: 5′ATAGCGGTG GACCCATGTCT-3′; MCM4: F: 5′-CCTCATTGGTAAAGGGC TAGAG-3′ and R: 5′-TAGCCAGGGTGACAGAGTAA-3′; MC M7: F:5′-CCAGGAGATGAAGATGCAAGAA-3′ and R: 5′-GG GCAATCCTTGTGTTCTCT-3′; BCL2L: F: 5′-TCAGGCTGCTT GGGATAAAG-3′ and R: 5′-AGGCTTCTGGAGGACATTTG-3′.

### RNA-Seq Analysis

Total cellular RNA was isolated using the TRIzol reagent. Briefly, mRNA was reversed to cDNA for constructing the library. Then, the cDNA library was measured by RNA sequencing. The raw reads were filtered, and clean reads were mapped using Bowtie2 and HISAT. The gene expression level (FPKM) was calculated according to the RSEM, and the data were analyzed.

### Western Blot

B-cell acute lymphocytic leukemia cell lines were seeded at a density of 10^6^ cells/ml and treated with drugs. Total cells were collected and lysed in SDS sample buffer. The primary antibodies used were as follows: ACTIN, GAPDH, TUBULIN as internal control (1:5000; Hua An Biotechnology, Hangzhou, China), CDK9 (1:1000; CST), Phospho-Rpb1 CTD (Ser2) (#13499, 1:1000; CST), Phospho-Rpb1 CTD (Ser5) (#13523, 1:1000; CST), Rpb1 CTD (1:1000; AM39097), BCL2 (#3212; 1:1000; Abcam), caspase 3 (ab13847, 1:1000; Abcam), cleaved caspase 3 (#9661, 1:1000; CST), GLUT1 (AF1015, 1:500; Beyotime), HK2 (#2867, 1:1000; CST), LDHA (#3582, 1:1000; CST), c-Myc (#5605, 1:1000; CST, Danvers, MA, United States), and Flag (0912-1, 1:1000; Hua An Biotechnology, Hangzhou, China). After incubation with the fluorescence-labeled secondary antibody, fluorescence signals were analyzed using the Odyssey system (LI-COR Biosciences, Lincoln, NE, United States).

### Apoptosis Analysis

Cell apoptosis was measured using the Annexin-V apoptosis detection kit (BD Bioscience, San Jose, CA, United States) according to the manufacturer’s protocol. The percentage of Annexin-V positive cells were detected by flow cytometry (BD Biosciences), and the data were analyzed using the FlowJo Version 10.0 software.

### Cell Proliferation Analysis

After the cells were treated with drugs for 24 h, EdU was added into the cells and incubated for 2 h. Cell proliferation was conducted using the Click-iT EdU flow cytometry assay kit (Beyotime) according to the manufacturer’s protocol. Then, the stained cells were analyzed by flow cytometer.

### Glucose Uptake Assay

The glucose uptake ability of the cells was detected by incubating with 2-NBDG (Invitrogen). Briefly, the cells were harvested and washed with PBS. Then, fluorescent 2-NBDG was added to the cells, which were then incubated at 37°C for 30 min in 5% CO2 incubator. After centrifugation, all media were removed and washed with PBS once, and the samples were analyzed using a flow cytometer.

### Extracellular Acidification Rate (EACR)

Metabolic flux analysis with a XF Glycolytic Stress Test Kit (#103017-100, Seahorse Bioscience) was performed using a Seahorse XF 96 instruments (Seahorse Bioscience). An equal number of REH cells was plated and treated with inhibitor for 24 h. Cartridge was equilibrated overnight prior to the assay day. Exactly 5 × 10^5^ cells were changed to base media supplemented with 10 mM glucose, 1 μM oligomycin, and 50 mM 2-DG. Results were analyzed using GraphPad Prism. Basal glycolytic rate and glycolytic capacity were calculated according to the manufacturer’s instructions.

### Lactate Concentration Assay

Lactate was quantified using Glycolysis cell-based Assay Kit (Cayman) according to the manufacturer’s protocol. Briefly, 5 × 10^4^ cells were cultured in RPMI-1640 medium supplemented with 0.25% fetal bovine serum (Gibco) for 24 h in 96 wells, and then treated with drugs for 24 h. Exactly 10 μl of culture supernatant was added to the lactate assay buffer. The reaction was incubated for 30 min at room temperature. The absorbance at 490 nm was assessed using a microplate reader.

### Measurement of Metabolic Indicators

Cell lines were seeded in RPMI 1640 complete medium with drugs. Cells were incubated with metabolic dyes, such as mitochondrial membrane potential probe MitoTrackerTM orange (Life Technologies, M7511), total ROS probe DCFDA (Life Technologies, C369). The samples were analyzed by flow cytometry.

### Metabolite Analysis

A total number of 2 × 10^6^ cells were harvested and washed with pre-cooled PBS. Cells were cracked in ice-cold 80% methanol and centrifuged at 1,500 rpm for 10 min to obtain the supernatant. Finally, the supernatant was detected by liquid chromatography–tandem mass spectrometry (LC-MS/MS).

### Construction of Overexpression Stable Cell Lines

The c-Myc overexpressed and vector plasmids were gifted from Dr. Li T (SCMC, China). The package and concentration of virus were conducted as previously reported (Wu et al., 2018). REH cells were cultured with the enriched viral medium for 48 h, and then selected with puromycin to construct the stable cell lines for 48 h. The overexpression efficiency was verified by Western blot analysis.

### Statistical Analysis

Statistical analysis was conducted using GraphPad Prism 7.0 (GraphPad Software). Data were presented as mean ± SD of three independent experiments. Differences between samples were analyzed using two-tailed Student’s *t*-test. Results with values of *P* < 0.05 were considered statistically significant.

## Results

### CDK9 Inhibitor SNS-032 Induces the Apoptosis of B-ALL Cell Lines *in vitro*

**FIGURE 1:**
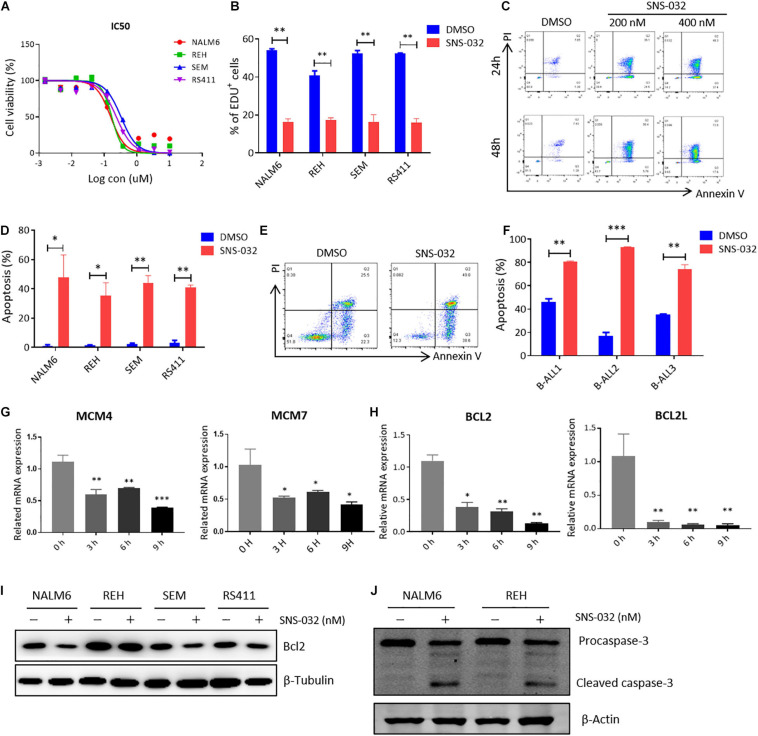
SNS-032 treatment inhibits proliferation and induces apoptosis in B-ALL cells. **(A)** Drug sensitivity assay of NALM6, REH, SEM, and RS4;11 cell lines. B-ALL cells were treated with a gradient concentration of SNS-032 for 72 h. **(B)** EdU-labeled cell cycle of B-ALL cell lines was analyzed by flow cytometry. **(C)** Annexin V and PI labeled cell apoptosis of REH cells was analyzed by flow cytometry. **(D)** Statistical analysis of the cell apoptosis rates of B-ALL cell lines. **(E)** Annexin V- and PI-labeled cell apoptosis of B-ALL patient sample was analyzed by flow cytometry. **(F)** Statistical analysis of cell apoptosis rates in three patients. **(G,H)** The DNA replication and anti-apoptosis genes of B-ALL cells were measured by qRT-PCR. **(I,J)** The anti-apoptosis and apoptosis proteins of B-ALL cells were detected by Western blot analysis. B-ALL cells were treated with SNS-032 (200 nM for NALM6, 200 nM for REH, 350 nM for SEM, 250 nM for RS411, and 200 nM for primary B-ALL cells) for 24 h. Values were shown as mean ± SEM. ^∗^*p* < 0.05, ^∗∗^*p* < 0.01, and ^∗∗∗^*p* < 0.001.

To determine the cytotoxic effects of CDK9 inhibitor (CDK9i) on B-ALL cells, we detected the cell viability in B-ALL cell lines after treatment with a gradient concentration of SNS-032 for 72 h. The IC50 values of NALM6, REH, SEM, and RS411 were 200, 200, 350, and 250 nM, respectively (Figure 1A). We then measured the cell proliferation by staining with EdU and found that EdU-positive B-ALL cells dramatically decreased after treatment with SNS-032 for 24 h (Figure 1B), and this result is consistent with a previous report in AML (Wang et al., 2019). Then, we detected the apoptosis of B-ALL cells after SNS-032 treatment for 24 and 48 h and found that the apoptotic rates significantly increased in all of B-ALL cell lines (Figures 1C,D), indicating that SNS-032 induces the cell death of B-ALL. We also determined the cell apoptosis in samples from patients with B-ALL and confirmed that the apoptotic rates increased in SNS-032-treated sample compared with that of DMSO-treated sample (Figures 1E,F and Supplementary Table 1). Additionally, the cell cycle and apoptosis of human normal peripheral blood mononuclear cells (PBMCs) were measured by flow cytometry, and the results showed that SNS-032, to a certain extent, induced apoptosis of PBMC, but did not affect the cell cycle of PBMC (Supplementary Figures 1A,B), indicating that SNS-032 may lead to side effects, such as myelosuppression. Furthermore, we used qRT-PCR to detect the expression of cell proliferation- and apoptosis-related genes. SNS-032 remarkably downregulated the expression of cell proliferation genes, such as MCM4 and MCM7, and the expression of anti-apoptosis genes, such as Bcl2 and BCL2L (Figures 1G,H). Moreover, Western blot was used to evaluate the protein expression of Bcl-2 and cleaved Caspase 3. The data displayed that Bcl-2 was down-regulated, and cleaved caspase 3 was markedly up-regulated in SNS-032-treated B-ALL cells (Figures 1I,J), indicating that SNS-032 activates the cell apoptosis signal pathway of B-ALL. The cytotoxic effects of SNS-032 were confirmed through degraded CDK9 protein, which inhibited phosphorylating serine 2 and 5 in the CTD of RNA Pol II in B-ALL cells (Supplementary Figure 1C). Taken together, these data suggested that CDK9i induces cell apoptosis and suppresses cell proliferation of B-ALL *in vitro*.

### SNS-032 Perturbs the Cellular Metabolic Pathways of B-ALL Cells *in vitro*

**FIGURE 2:**
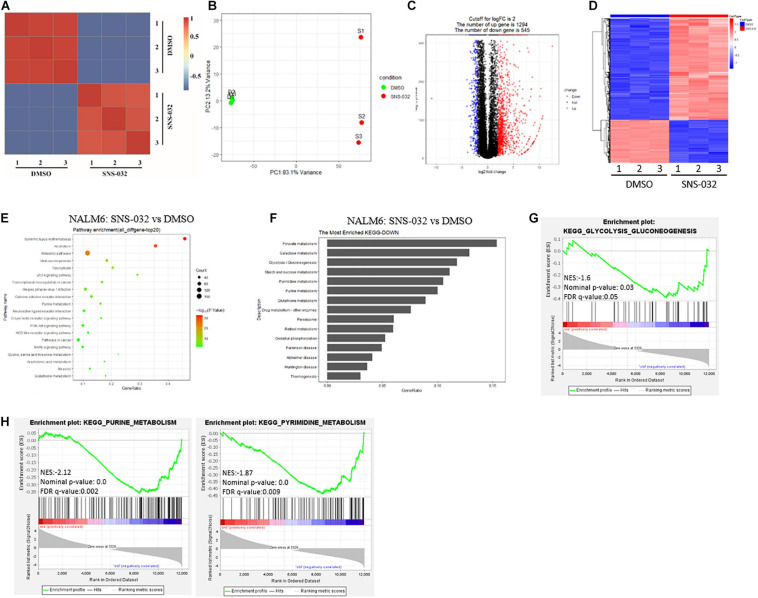
Effect of SNS-032 treatment on the cellular metabolic pathways of B-ALL cells. **(A,B)** Correlation analysis and principal component analysis of the RNA-seq data from SNS-032-treated versus DMSO-treated REH cells. **(C,D)** Volcano plot and heatmap of differentially expressed genes by a log2-fold change ≥ 2.0 or ≤ −2.0 (padj < 0.001) in SNS-032-treated versus DMSO-treated REH cells. **(E)** Pathway analysis of up- and downregulated genes (padj < 0.001) in SNS-032-treated versus DMSO-treated REH cells. **(F)** Pathway analysis of downregulated metabolic genes (padj < 0.001) in SNS-032-treated versus DMSO-treated REH cells. **(G,H)** Gene set enrichment analysis of the genes related to purine, pyrimidine, and glycolysis.

Tumor cells reprogram their metabolism from catabolism to anabolism to prompt cells enter the cell cycle and fuel cell proliferation (Faubert et al., 2020). CDK9 inhibition promotes prostate cancer cells switch to fatty acid oxidation by inducing metabolic stress (Itkonen et al., 2019). However, the inhibitory effect of CDK9i on the energy metabolism of B-ALL cells remains unclear. To address this question, RNA sequencing (RNA-seq) was performed in B-ALL cells after treated with SNS-032 or DMSO for 24 h. The SNS-032- and DMSO-treated cell populations were clustered by correlation analysis and principal component analysis (Figures 2A,B). The transcript profile of SNS-032-treated cells was globally changed, where 1,294 genes were upregulated and 545 genes were downregulated compared with those of DMSO-treated cells (Figures 2C,D). In addition, the up- and downregulated genes were analyzed by Kyoto Encyclopedia of Genes and Genomes (KEGG) analysis. We found that SNS-032 significantly down-regulated the mRNA expression of p53 signaling pathway, PI3K-Akt signaling pathway, and metabolic pathways (Figure 2E). We further analyzed the metabolic pathways and found that pyruvate metabolism, glycolysis/gluconeogenesis, purine/pyrimidine metabolism and oxidative phosphorylation were greatly changed after treatment with SNS-032 (Figures 2F–H). Altogether, these findings indicated that CDK9i perturbs the glycolytic metabolism of B-ALL cells *in vitro*.

### SNS-032 Prompts the Apoptosis of B-ALL Cells by Inhibiting Glycolysis

**FIGURE 3:**
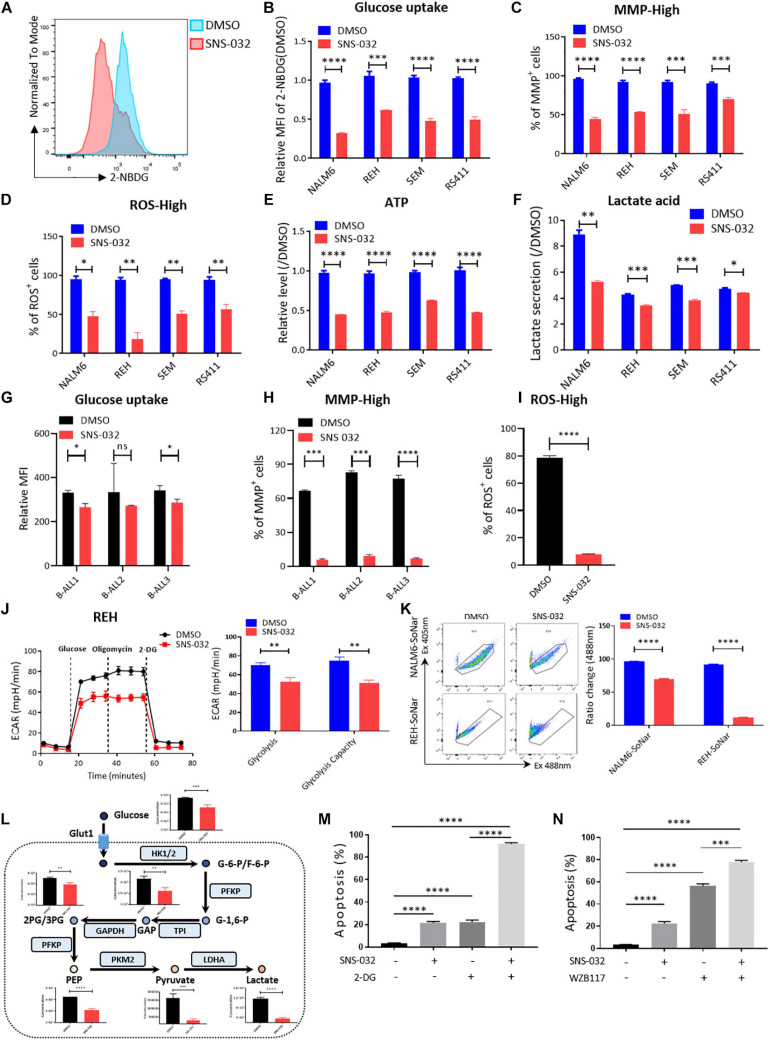
SNS-032 treatment downregulates glucose metabolism in B-ALL cells. **(A)** The glucose uptake of NALM6, REH, SEM, and RS411 cells was detected by flow cytometry. **(B)** Statistical analysis of the glucose uptake of B-ALL cell lines. **(C,D)** The mitochondrial membrane potential (MMP) and total ROS of B-ALL cells were detected by flow cytometry. **(E)** The quantification of intracellular ATP in B-ALL cells was assessed using the ATP determination kit. **(F)** Quantification of intracellular lactate in B-ALL cell lines using the lactate assay kit. **(G)** The glucose uptake of primary B-ALL cells was detected by flow cytometry. **(H,I)** The MMP and total ROS of primary B-ALL cells were detected by flow cytometry. **(J)** Detection of ECAR in REH cell using Seahorse XF 96. **(K)** The SoNar-low or -high cells of B-ALL cells were measured by flow cytometry with 405 and 488 nm excitation. **(L)** Schematic map of glycolytic metabolism and the metabolic intermediates of glycolysis measured by LC-MS/MS. **(M,N)** Cell apoptosis of SNS-032-treated REH cells analyzed by flow cytometry after co-treatment with 2-DG (1 mM) and WZB117 (10 nM) for 24 h. Values were shown as mean ± SEM. ^∗^*p* < 0.05, ^∗∗^*p* < 0.01, ^∗∗∗^*p* < 0.001 and ^****^p < 0.0001.

To clarify the effects of CDK9i on the glycolytic metabolism of B-ALL cells, we detected the glucose uptake of B-ALL cells by incubating fluorescence-labeled 2-deoxy-glucose analog (2-NBDG). SNS-032 treatment remarkably reduced the glucose uptake activities in B-ALL cells (Figures 3A,B). In addition, the mitochondrial membrane potential (MMP), ATP content, total reactive oxygen species (ROS) and intracellular lactate concentration were markedly decreased in all four cell lines after SNS-032 treatment (Figures 3C–F). The decreased of glucose uptake, MMP and total ROS were confirmed in SNS-032-treated primary B-ALL cells (Figures 3G–I). To directly determine the glycolytic capacity of B-ALL cells, the extracellular acidification rate (ECAR) was measured by Seahorse. We uncovered that SNS-032 treatment dramatically inhibited the glycolysis of B-ALL cells (Figure 3J). To further prove whether SNS-032 suppresses glycolysis, we used SoNar, a metabolic sensor, to monitor the dynamic of metabolic change and found that SoNar-high cells prefer glycolysis (Zhao et al., 2015; Zou et al., 2018). The alteration of the ratios in SoNar B-ALL cells was easily tested by flow cytometry. The results displayed that the ratios of SoNar-high cells notably decreased in SNS-032-treated cells (Figure 3K). Moreover, the intermediates of glycolysis in SNS-032-treated B-ALL cells were measured by LC-MS/MS, and the data revealed that the levels of metabolic intermediates of glycolysis, such as glucose-6-phosphate, glyceraldehyde-3-phosphate, pyruvate, and lactate, considerably dropped in SNS-032-treated B-ALL cells (Figure 3L). Hence, SNS-032 restrains the glycolysis of B-ALL cells *in vitro*.

Metabolic shift occurs in the survival, invasion, and metastasis of cancer cells. Glycolysis, which is the main energy source of tumor cells, is inextricably coupled with cell proliferation and death (Buchakjian and Kornbluth, 2010; Kishton et al., 2016). To testify that SNS-032 results in the cell death of B-ALL cells by restraining glycolysis, cell apoptosis after co-treatment with a glycolysis inhibitor, 2-Deoxy-D-glucose (2-DG), was measured by flow cytometry. We uncloaked that the cell apoptosis induced by SNS-032 was markedly enhanced in 2-DG co-treated cells (Figure 3M). Additionally, the cell apoptosis induced by SNS-032 was significantly improved in GLUT1 inhibitor WZB117 co-treated cells (Figure 3N). Overall, these results indicated that SNS-032 leads to the apoptosis of B-ALL cells by partially inhibiting glycolysis.

### CDK9 Inhibitor AZD4573 Facilitates the Apoptosis of B-ALL Cells by Inhibiting Glycolysis

**FIGURE 4:**
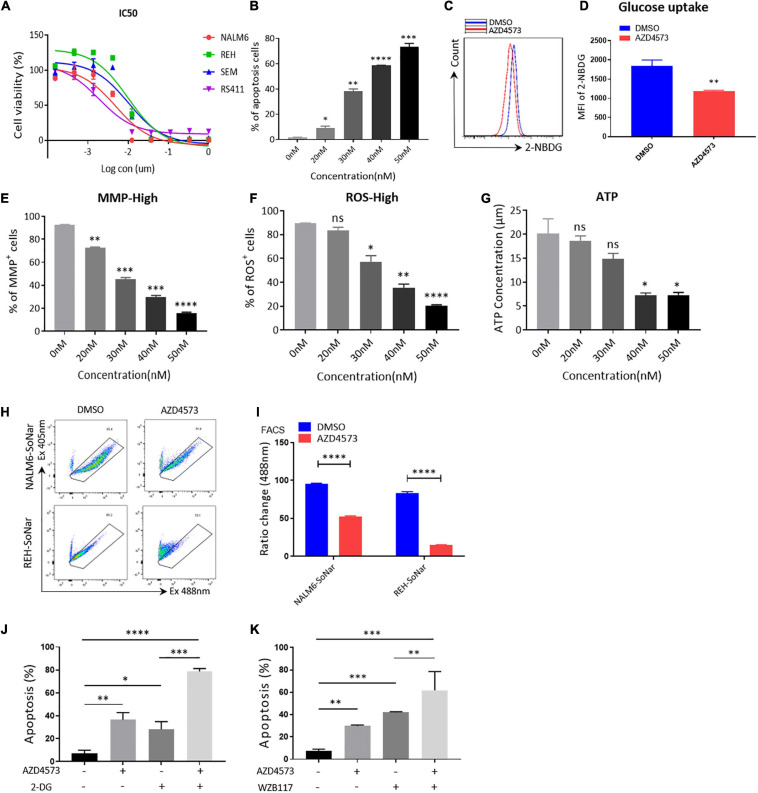
AZD4573 treatment results in the cell death of B-ALL cell lines. **(A)** Drug sensitivity assay of NALM6, REH, SEM, and RS411 cell lines. B-ALL cells were treated with a gradient concentration of AZD4573 for 72 h. **(B)** Annexin V- and PI-labeled cell apoptosis of REH cells was analyzed by flow cytometry. **(C)** The glucose uptake of REH cells was detected by flow cytometry. **(D)** Statistical analysis of glucose uptake of REH cells. **(E,F)** MMP and total ROS of REH cells was analyzed by flow cytometry. **(G)** The quantification of intracellular ATP in B-ALL cells was assessed using the ATP determination kit. **(H)** The SoNar-low or -high cells of B-ALL cells were measured by flow cytometry with 405 and 488 nm excitation. **(I)** Statistical analysis of SoNar-high B-ALL cells. **(J,K)** Cell apoptosis of AZD4573-treated NALM6 cells analyzed by flow cytometry after co-treatment with 2-DG (1 mM) and WZB117 (10 nM) for 24 h. B-ALL cells were treated with AZD4573 for 24 h. Values were shown as mean ± SEM. ^∗^*p* < 0.05, ^∗∗^*p* < 0.01, ^∗∗∗^*p* < 0.001 and ^****^p < 0.0001.

To further confirm that CDK9i restrains the glycolytic metabolism of B-ALL cells *in vitro*, we used AZD4573, a highly selective CDK9 inhibitor, to evaluate the effects of CDK9i on the cell apoptosis of B-ALL cells. As shown in Figure 4A, the IC50 values of NALM6, REH, SEM, and RS411 are 5, 10, 10, and 1 nM, respectively. In addition, we found that AZD4573 induces the apoptosis of REH cells in a dose-dependent manner (Figure 4B). Meanwhile, AZD4573-treated REH cells exhibited lower glucose uptake activities compared with those of DMSO-treated cells (Figures 4C,D). Furthermore, AZD4573 treatment decreased levels of MMP, ROS and the ATP content in a dose-dependent manner (Figures 4E–G). Moreover, AZD4573 treatment reduced the ratios of SoNar-high cells in B-ALL cells (Figures 4H,I), indicating that AZD4573 restrains the glycolysis of B-ALL cells *in vitro*. More importantly, the cell apoptosis induced by AZD4573 was increased in cells co-treated with glycolysis inhibitors 2-DG and WZB117 (Figures 4J,K). We also confirmed that AZD4573 resulted in the glycolysis inhibition of B-ALL cells by degrading CDK9 and phosphorylating serine 2 and 5 in the CTD of RNA Pol II (Supplementary Figure 2). Hence, CDK9 inhibitors induce cell apoptosis by partially suppressing the glycolysis of B-ALL cells *in vitro*.

### CDK9i Curbs the Glycolysis of B-ALL Cells by Downregulating the Expression of Metabolic Enzymes

**FIGURE 5:**
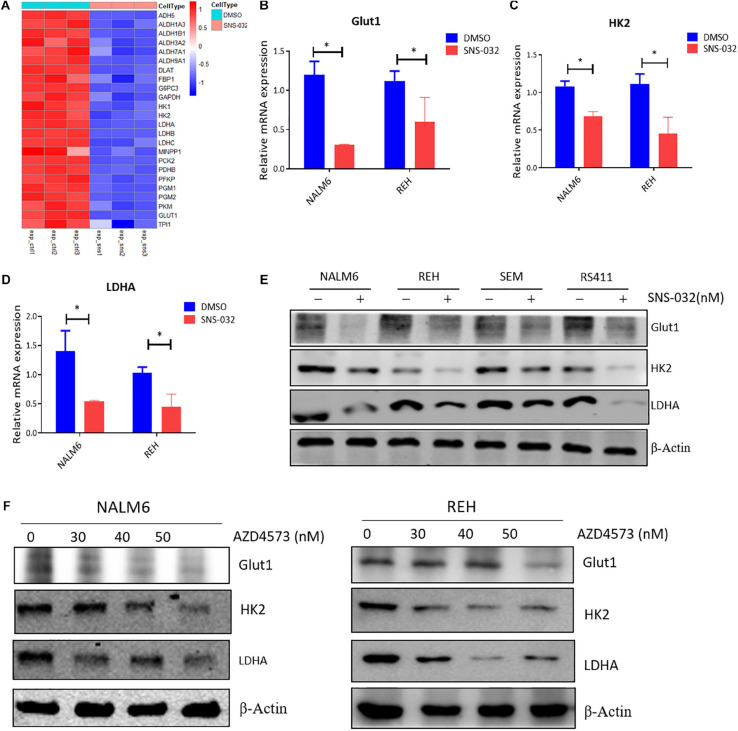
CDK9i treatment alters the expression of metabolic enzymes in B-ALL cells. **(A)** Heatmap of glycolysis-related genes in REH cells analyzed by RNA-seq. **(B–D)** Relative mRNA expression levels of GLUT1, HK2, and LDHA measured by qRT-PCR in NALM6 and REH cells after treatment with SNS-032 for 24 h. **(E)** Protein expression levels of GLUT1, HK2, and LDHA in B-ALL cells detected by Western blot analysis after treatment with SNS-032 for 24 h. **(F)** Protein expression levels of GLUT1, HK2, and LDHA in NALM6 and REH cells detected by Western blot after treatment with AZD4573 for 24 h. Values were shown as mean ± SEM. ^∗^*p* < 0.05.

As the initial step in glucose metabolism, glycolysis consists of several reactions that are involved in several key rate-limiting enzymes, such as hexokinase (HK), phosphofructokinase (PFK), and pyruvate kinase (PK) (Faubert et al., 2020). The RNA-seq data was re-analyzed to prove whether CDK9i suppressed the glycolysis of B-ALL cells by down-regulating the expression of metabolic enzymes. We discovered that SNS-032 remarkably down-regulated the key rate-limiting enzymes of glycolysis, such as GLUT1, HK2, and LDHA (Figure 5A). We performed qRT-PCR to validate the expression levels of glycolysis-related enzymes, and the results exhibited that SNS-032 dramatically downregulated the expression of GLUT1, HK2, and LDHA (Figures 5B–D). We then detected the protein expression levels of the rate-limiting enzymes in the glycolytic pathway. The results exhibited that SNS-032 markedly downregulated the expression levels of GLUT1, HK2, and LDHA (Figure 5E). Moreover, AZD4573 downregulated the expression levels of GLUT1, HK2, and LDHA (Figure 5F). These findings indicated that CDK9i restrains the glycolysis of B-ALL cells by reducing the expression of metabolic enzymes.

### CDK9i Engenders the Cell Apoptosis of B-ALL by Suppressing c-Myc-Mediated Glycolysis

**FIGURE 6:**
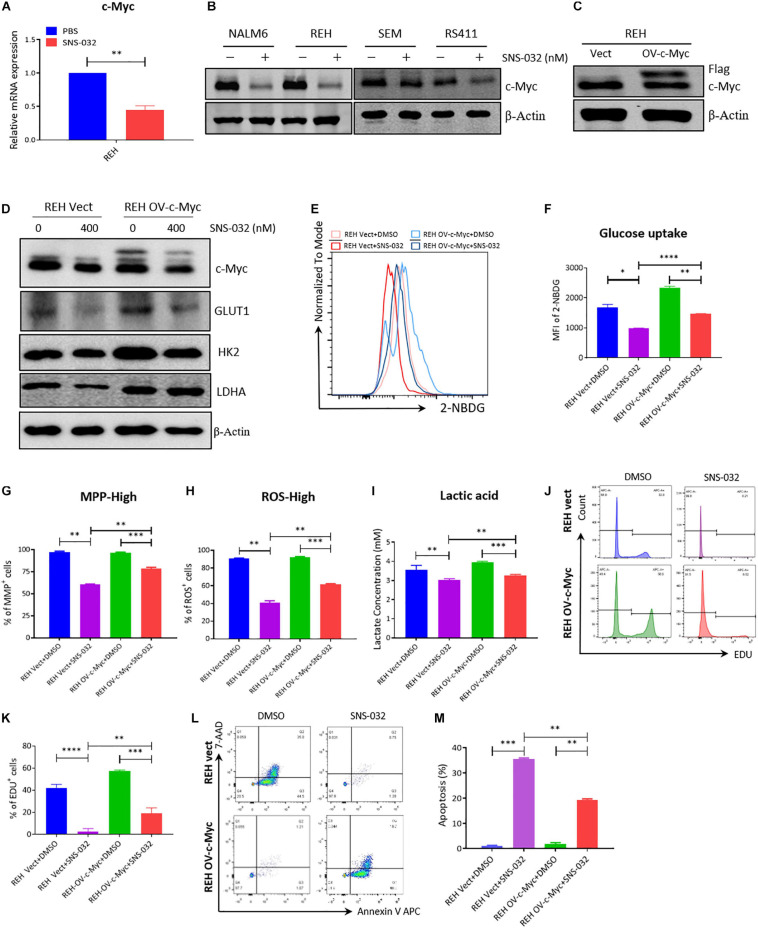
Overexpressed c-Myc in B-ALL cells rescues CDK9i induced cell apoptosis. **(A)** Relative mRNA expression levels of c-Myc in REH cells measured by qRT-PCR. **(B)** Protein expression of c-Myc in B-ALL cells determined by Western blot analysis. **(C)** Overexpressed level of c-Myc in REH cells detected by Western blot analysis. **(D)** Levels of HK2 and LDHA in c-Myc-overexpressing REH cells tested by Western blot analysis after intervention with SNS-032 at 0 and 400 nM. **(E)** Glucose uptake of vector and c-Myc-overexpressing REH cells was analyzed by flow cytometry. **(F)** Statistical analysis of glucose uptake of REH cells. **(G,H)** MMP and total ROS of vector and c-Myc-overexpressing REH cells were analyzed by flow cytometry. **(I)** Quantification of intracellular lactate in vector and c-Myc-overexpressing REH cells using the lactate assay kit. **(J)** EdU-labeled cell cycle of c-Myc-overexpressing REH cells analyzed by flow cytometry. **(K)** Statistical analysis of EdU-positive REH cells. **(L)** Annexin V- and 7-AAD-labeled cell apoptosis of c-Myc-overexpressing REH cells was analyzed by flow cytometry. **(M)** Statistical analysis of cell apoptosis rates of B-ALL cells. B-ALL cells were treated with SNS-032 for 24 h. Values were shown as mean ± SEM. ^∗^*p* < 0.05, ^∗∗^*p* < 0.01, ^∗∗∗^*p* < 0.001 and ^****^p < 0.0001.

CDK9 inhibition prevents productive transcription and downregulates the expression of many genes, such as c-Myc and Mcl-1 (Boffo et al., 2018). C-Myc stimulates the anabolism of cancer cells by directly modulates the expression of several glycolysis genes, such as GLUT1, PKM2, and LDHA (Liang et al., 2016; Fang et al., 2019). We deduced that CDK9i induces cell apoptosis by downregulating the expression of c-Myc-mediated glycolysis genes. To prove this hypothesis, we first confirmed that SNS-032 suppressed the mRNA and the protein expression of c-Myc in B-ALL cells (Figures 6A,B). To check whether the SNS-032-induced reduction of glycolysis in leukemia cells is mediated by c-Myc, we over-expressed c-Myc on REH cells by lentivirus infection. The overexpressed c-Myc protein in REH cells was verified by Western blot (Figure 6C). SNS-032 treatment did not affect the overexpression of c-Myc (Figure 6D). We also demonstrated that the glycolytic enzymes were reversed by overexpressing c-Myc upon treatment with SNS-032 (Figure 6D). Furthermore, the levels of glucose uptake, MMP, total ROS, and intracellular lactate were partially rescued in c-Myc-overexpressing B-ALL cells after treatment with SNS-032 (Figures 6E–I), implying that CDK9i blocked the glycolysis of B-ALL cells by reducing c-Myc expression. Additionally, EdU-positive proliferating cells were evidently restored in c-Myc-overexpressing B-ALL cells after intervention with SNS-032 (Figures 6J,K). More importantly, the cell apoptosis was abolished in c-Myc-overexpressing B-ALL cells after intervention with SNS-032 (Figures 6L,M). These data suggested that CDK9i induces the apoptosis of B-ALL cells by partly inhibiting c-Myc-mediated glycolytic gene expression.

## Discussion

Leukemic cells infiltrated and destructed the bone marrow, and then disrupted the normal hematopoiesis, leading to the death of patients. Through multi-modal combination chemotherapy or hematopoietic stem cell transplantation, the 5-year overall survival rate of patients with childhood B-ALL has reached over 80% (Malard and Mohty, 2020). However, a proportion of B-ALL patients is not sensitive to chemotherapy and still suffer from relapse, leading to treatment failure (Teachey and Pui, 2019; Malard and Mohty, 2020). Therefore, new drugs should be developed to improve the treatment rates and overcome drug resistance and B-ALL relapse. In preclinical studies, CDK9 inhibitors have demonstrated anti-tumor effects in many different types of tumor (Morales and Giordano, 2016). SNS-032, a selective and potent inhibitor of CDK 2, 7, and 9 and AZD4573, a highly selective inhibitor of CDK9, exhibited the inhibitory effects of hematological malignant cell lines *in vitro* and clinical therapeutic activity in patients with MM and CLL (Tong et al., 2010; Walsby et al., 2011). A study reported that CDK9 is overexpressed in B-ALL through hub analysis. They also observed that the RNA and protein expression levels of CDK9 were high in MOLT4 and REH leukemic cell lines in the Human Protein Atlas database. These data indicated that CDK9 could serve as potential biomarkers and predictors of leukemogenesis in B-ALL (Jayaraman et al., 2015). In the present study, we found that SNS-032 and AZD4573 induced cell apoptosis of both B-ALL cell lines and patients’ samples in a dose- and time-dependent manner *in vitro*. Notably, the IC50 values of AZD4573 were lower than those of SNS-032, indicating that CDK9 is a highly selective inhibitor with high potential in the treatment of B-ALL. Moreover, our data indicated that triggering programmed cell death, resulting in B-ALL cell apoptosis, is the key to the treatment with CDK9 inhibitors. Thus, CDK9 could also serve as a novel target for B-ALL therapy.

Enhanced glycolysis is prerequisites for the rapid proliferation of tumor cells (Faubert et al., 2020). CDKs affect the catalytic activity of metabolic rate-enzymes and modulate the cell cycle arrest and apoptosis of tumor cells (Wang et al., 2017; Icard et al., 2019). However, the effect of CDK9 inhibitors on the cellular metabolism of B-ALL cells is unknown. In the study, we first observed that SNS-032 perturbs the cellular metabolic pathways of B-ALL cells, especially the glycolytic pathway. Therefore, we inferred that CDK9 inhibitors induced the cell apoptosis of B-ALL cells by suppressing glycolysis. By using Seahorse and LC-MS/MS to detect the metabolism of drug-treated cells, we uncovered that SNS-032 can significantly restrain the glycolysis of B-ALL cells by repressing glucose metabolism, thus reducing the metabolic intermediates, such as ATP and lactate, which are the energy sources and main materials for cellular anabolism (Ganapathy-Kanniappan, 2018; Abdel-Wahab et al., 2019). To further confirm our results, we used SoNar probe to dynamically detect the metabolic change and revealed that the ratios of SoNar-high cells significantly decreased upon treatment with SNS-032 and AZD4573, suggesting that CDK9 inhibitors suppressed the glycolysis of B-ALL cells. The results of RNA-seq indicated that SNS-032 restrained the glycolytic process by downregulating the expression of key enzymes, such as HK2, PFK, and LDHA. Moreover, the glycolysis inhibitors WZB117 and 2-DG enhanced the cell apoptosis of B-ALL cells induced by SNS-032 and AZD4573, suggesting that CDK9 inhibitors resulted in the apoptosis of B-ALL by partially inhibiting glycolysis. CDK9 inhibition promotes prostate cancer cells switch to fatty acid oxidation by inducing metabolic stress (Itkonen et al., 2019). In the present study, we did not observe the fatty acid metabolism on the top of SNS-032-inhibited pathway. Notably, SNS-032 not only affected glycolysis but also the purine/pyrimidine metabolism and oxidative phosphorylation of B-ALL cells, thus requiring further mechanism exploration.

As CDK9 inhibitors, SNS-032 and AZD4573 stop the gene transcription and results in the downregulated expression of a large proportion of genes, such as c-Myc and Mcl-1 (Boffo et al., 2018). Based on the results of RNA-seq, we found that SNS-032 dramatically reduced the expression of c-Myc. Moreover, the protein level of c-Myc decreased in B-ALL cells after SNS-032 and AZD4573 treatment. The metabolic reprogramming of tumor cells attributes to the regulation of target genes expression mediated by c-Myc (Dang et al., 2009; Hsieh et al., 2015). Thus, we infer that glycolysis is inhibited by CDK9 inhibitors because of the reduction of c-Myc level. The rescue experiment was performed to observed that the therapeutic effect of CDK9 inhibitors by overexpressing c-Myc in B-ALL cells. The cell apoptosis was abolished in c-Myc-overexpressing B-ALL cells after treatment with CDK9 inhibitors, accompanied by the relief of glycolysis, suggesting that the inhibitory effect in glycolysis of CDK9 inhibitors was mediated by downregulating c-Myc. This finding can be supported by flow cytometry with FITC Annexin V and PI staining that c-Myc overexpression could suppress SNS-032-induced apoptosis in REH cells. Meanwhile, the overexpression of c-Myc in REH cell enhanced glucose utilization, lactate production, and cell proliferation and inhibited apoptosis. Many glucose metabolism genes, such as GLUT1, HK2, PFKM, and LDHA, were documented to be directly regulated by c-Myc (Miller et al., 2012). To further explore the potential mechanism of c-Myc-mediated apoptosis upon SNS-032 treatment, we examined the protein expression of glycolysis-related gene. Our data demonstrated that the overexpression of c-Myc upregulated the mRNA and protein expression of GLUT1, HK2, and LDHA, thereby increasing glycolysis in REH cells. In addition, the expression of glycolysis-related genes was inversely correlated with the c-Myc expression level in the REH cell line treated with SNS-032, suggesting that c-Myc exerted an antagonistic effect on SNS-032-induced apoptosis by regulating glycolytic-related protein expression. In our study, whether c-Myc can reverse SNS-032 induced apoptosis by directly binding to the promoters of glycolytic genes requires further exploration.

## Conclusion

**FIGURE 7:**
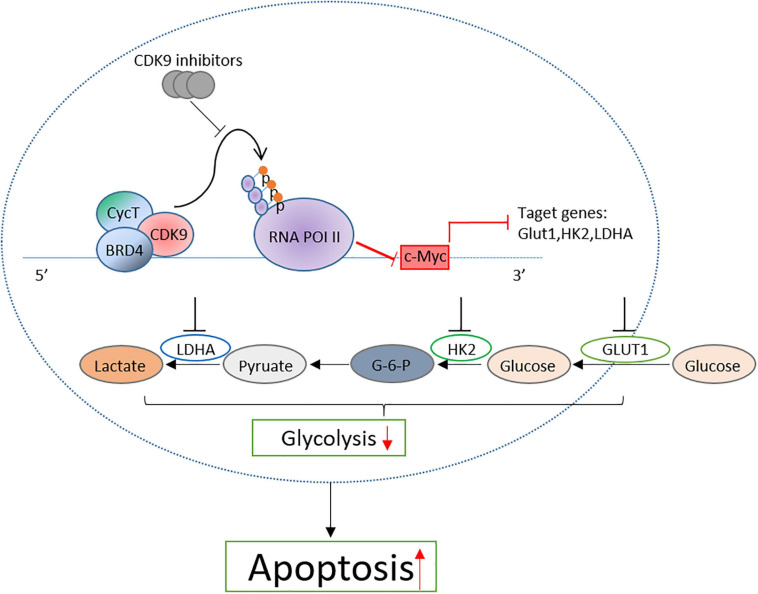
Proposal model of CDK inhibition induced the apoptosis of B-ALL cells by suppressing c-Myc mediated glycolysis.

Taken together, by detecting the therapeutic effect of CDK9 inhibitors on B-ALL cell lines, we confirmed that CDK9 inhibitors induced the cell apoptosis of leukemic cells by inhibiting the c-Myc-mediated glycolysis and revealed the mechanism of CDK9 inhibitors in the treatment of B-ALL (Figure 7). This study provides a new treatment strategy for B-ALL in clinical practice.

## Data Availability Statement

The data presented in the study are deposited in the GEO repository, accession number GSE166339.

## Ethics Statement

The studies involving human participants were reviewed and approved by the SCMC Ethics Committee. The patients/participants provided their written informed consent to participate in this study.

## Author Contributions

C-WD, GY, and SG designed the study, analyzed and interpreted the data, and wrote the manuscript. W-LH and TA performed the experiments, and analyzed and interpreted the data. JX analyzed the RNA-seq data. HZ, W-WZ, NZ, R-YS, M-HL, J-MZ, and CJ performed the experiments. K-WL, KQ, and LC discussed the results and contributed to data interpretation. All authors read and approved the final manuscript.

## Conflict of Interest

The authors declare that the research was conducted in the absence of any commercial or financial relationships that could be construed as a potential conflict of interest.
