# Supplementary material for: CDK9 Inhibitor Induces the Apoptosis of B-Cell Acute Lymphocytic Leukemia by Inhibiting c-Myc-Mediated Glycolytic Metabolism
Source: Front Cell Dev Biol. 2021 Mar 4;9:641271. doi: 10.3389/fcell.2021.641271 (PMC7969802; doi:10.3389/fcell.2021.641271)
Supplement: Supplementary file 1 [file Data_Sheet_1.docx]

Supplementary Material

CDK9 inhibitor induces the apoptosis of acute lymphocytic leukemia by inhibiting c-Myc-mediated glycolytic metabolism

Wen-Li Huang, Tuersunayi Abudureheman, Jing Xia, Lei Chu, Hang Zhou, Wei-Wei Zheng, Neng Zhou, Rong-Yi Shi, Ming-Hao Li, Jian-Min Zhu, Kai Qing, Chao Ji, Kai-Wei Liang, Sa Guo, Gang Yin and Cai-Wen Duan

**Table S1**

Clinical data for primary ALLs

| Sample | Sample type | cytogenetics | Age at diagnosis | Gender | White cell count  (×10^9^ cells) |
| --- | --- | --- | --- | --- | --- |
| B-ALL1 | Primary | Fusion gene negative | 6 years | male | 43.2 |
| B-ALL2 | Primary | Fusion gene negative | 3 years | male | 58.5 |
| B-ALL3 | Primary | BCR/ABL | 10 years | famale | 126 |


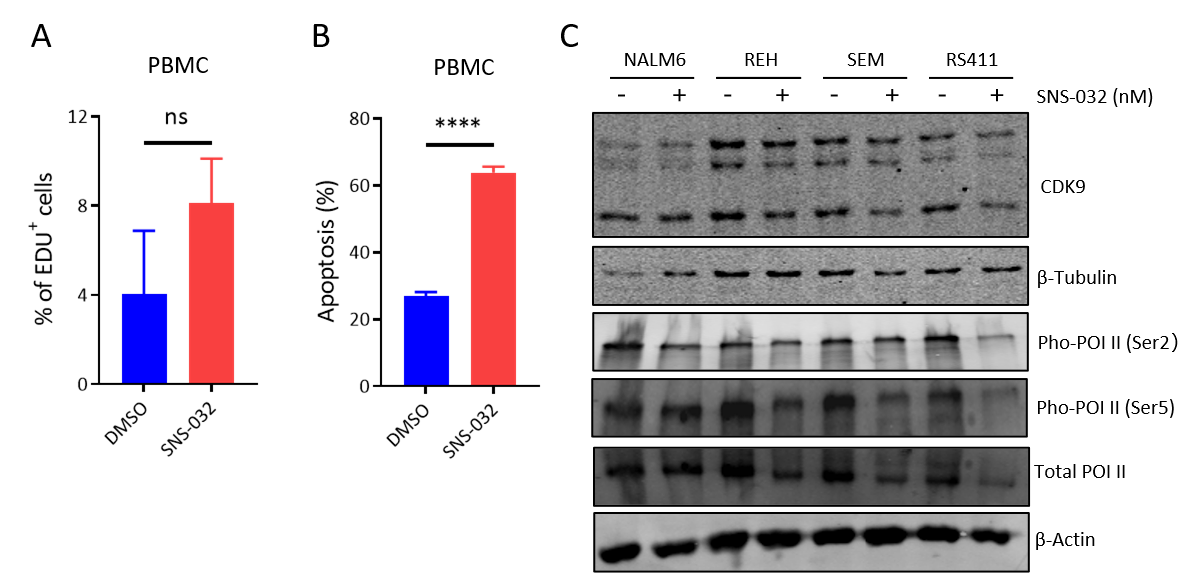


**Figure S1.** SNS-032 inhibits cell proliferation and induces apoptosis in B-ALL cells. (A) EdU-labeled cell cycle of human PBMCs was analyzed by flow cytometry after treatment with SNS-032 for 24 h. (B) Annexin V and PI labeled cell apoptosis of human PBMCs was analyzed by flow cytometry after treatment with SNS-032 for 24 h. (C) Protein expression levels of CDK9, Pho-POI II (Ser2), Pho-POI II (Ser5) and total POI II in B-ALL cell lines detected by Western blot after treatment with SNS-032 for 24 h.


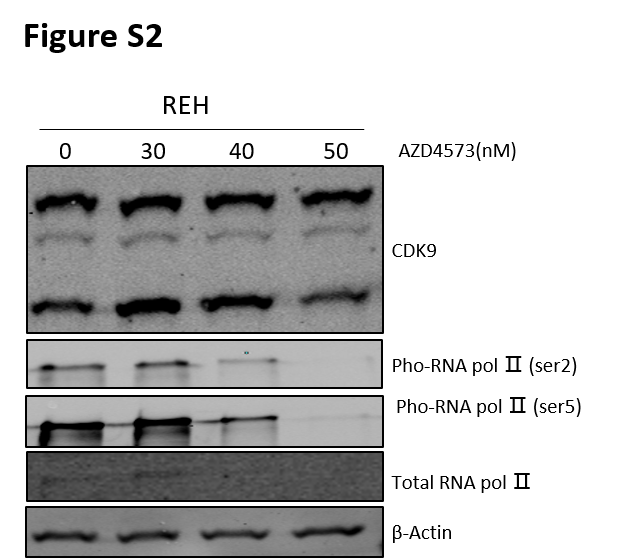


**Figure S2.** AZD4573 facilitates the apoptosis of B-ALL cells by inhibiting glycolysis. Protein expression levels of CDK9, Pho-POI II (Ser2), Pho-POI II (Ser5) and total POI II in REH cells detected by Western blot after treatment with AZD4573 for 24 h.
